# The effects of short messages encouraging prevention behaviors early in the COVID-19 pandemic

**DOI:** 10.1371/journal.pone.0284354

**Published:** 2023-04-14

**Authors:** Sophia L. Pink, Michael N. Stagnaro, James Chu, Joseph S. Mernyk, Jan G. Voelkel, Robb Willer

**Affiliations:** 1 Department of Sociology, Stanford University, Palo Alto, California, United States of America; 2 Sloan School of Management, Massachusetts Institute of Technology, Cambridge, Massachusetts, United States of America; 3 Department of Sociology, Columbia University, New York, New York, United States of America; University of Valencia: Universitat de Valencia, SPAIN

## Abstract

Effectively addressing public health crises like the COVID-19 pandemic requires persuading the mass public to change their behavior in significant ways. Many efforts to encourage behavior change–such as public service announcements, social media posts, and billboards–involve short, persuasive appeals, yet the effectiveness of these messages is unclear. Early in the COVID-19 pandemic, we tested whether short messages could increase intentions to comply with public health guidelines. To identify promising messages, we conducted two pretests (*n* = 1,596) in which participants rated the persuasiveness of 56 unique messages: 31 based on the persuasion and social influence literatures and 25 drawn from a pool of crowdsourced messages generated by online respondents. The four top-rated messages emphasized: (1) civic responsibility to reciprocate the sacrifices of health care workers, (2) caring for the elderly and vulnerable, (3) a specific, sympathetic victim, and (4) limited health care system capacity. We then conducted three well-powered, pre-registered experiments (total *n* = 3,719) testing whether these four top-rated messages, and a standard public health message based on language from the CDC, increased intentions to comply with public health guidelines, such as masking in public spaces. In Study 1, we found the four messages and the standard public health message significantly outperformed a null control. In Studies 2 and 3, we compared the effects of persuasive messages to the standard public health message, finding that none consistently out-performed the standard message. This is in line with other research showing minimal persuasive effects of short messages after the very early stages of the pandemic. Across our studies, we found that (1) short messages can increase intentions to comply with public health guidelines, but (2) short messages featuring persuasive techniques from the social science literature did not substantially outperform standard public health messages.

## Introduction

The coronavirus pandemic has infected more than 500 million people globally, claimed the lives of more than 6 million people [1], severely affected economic growth and employment in nearly every country, and changed everyday life as we know it. To ensure engagement in behaviors that reduce the spread of COVID-19, organizations and governments must generate public health messages that are compelling and persuasive. This is of contemporary importance, as novel variants of SARS-CoV-2 evade vaccines more effectively than previous strains, so behavioral compliance with public health guidelines–such as masking–has remained critically important [2]. In addition, future pandemics and other public health crises will require public health messaging campaigns.

A substantial body of work preceding the COVID-19 pandemic has tested whether short messages—such as those found on social media, public service announcements, or billboards—can impact people’s health behaviors [3–5]. Researchers across many disciplines have studied how to create messages that persuade people to engage in behaviors to protect their own and others’ health [6, 7]. Beyond public health, there is a vast, multidisciplinary literature on effective persuasive techniques [8]. Nevertheless, it is unclear the extent to which short messages were persuasive in the context of the early stages of the COVID-19 pandemic in the United States, and if they were, which persuasive strategies were most effective at promoting behavior change. The COVID-19 pandemic differs in many ways from recent public health crises—it has directly impacted every American’s life, required sudden and drastic behavior change, and occurred in a deeply polarized country with historically-low trust in media [9] and government [10]. People have been saturated with often conflicting messaging from a variety of news sources, the federal government, local and state governments, employers, schools, friends, and family. Consistent with this, views of the coronavirus pandemic have polarized along political lines [11], reported skepticism about the seriousness of COVID-19 is high, and conspiracy theories regarding its origins have proliferated [12]. Past research suggests that people would be most likely to be persuaded by messaging campaigns early in the pandemic, as messaging campaigns are less effective when issues have been salient for a longer time period [13–15].

### Short messages as a medium of persuasion

Short messages are one of the most important forms of persuasion available for agencies seeking to increase public health behavior compliance. There are many media through which persuasive appeals occur, but short messages, such as short radio scripts, video ads, text messages, or text on billboards, are ubiquitous. Research shows that short messages, which are scalable and consumable even to those with limited time to pay attention, are key for reaching the general public [16]. A meta-analysis of mass-media public health campaigns involving short messages found that messaging campaigns lead to approximately a 5% increase in the number of people performing the intended behavior [3]. Other reviews have also found that mass media campaigns have small to moderate effects on health behaviors [17], and that short messages can impact behaviors related to physical activity, sexual health, treatment-seeking, and smoking cessation [4, 5].

The field of social marketing [18, 19] has studied how to use communication strategies to persuade people to change their public health behaviors. Research on social media campaigns, has found that these campaigns can spread messaging, but there has been limited work on the extent to which social media campaigns change behavior [20, 21]. The effect of social media campaigns may also depend on audience skepticism—past work has found that consumers who are more skeptical about advertising like advertising less and are less responsive to advertising than those who are less skeptical [22].

However, the context of the COVID-19 pandemic has differed from the contexts of much of this past research in terms of scale, quantity of information people are receiving, and the politicization of the virus. Given this unique context, it is important to test whether short messages impacted prevention behaviors during the COVID-19 pandemic.

### Persuasive strategies

Many studies have been conducted across the behavioral and social sciences investigating which persuasive strategies used in short messages are most effective in encouraging public health behaviors. This literature spans many disciplines, making it difficult to review comprehensively. There are several messaging approaches and debates that are common to much of the literature about prevention behaviors.

First, some public health research has found that prosocial messages are more likely to change people’s behavior than messaging that appeals to self-interest [23, 24]. However, other work has shown that self-interested messages are just as effective, if not more effective, in contexts such as vaccination [25]. It is unclear which approach would be more effective in the context of the COVID-19 pandemic. Second, past research has found that stories of identifiable victims are more persuasive than statistics about larger groups, although there has been limited research on this approach in the context of public health behaviors [26]. Third, social proof—indicating that many other people are complying with guidelines—has been shown to be effective for prompting behavior change, although much of the behavior change research has been done in the context of energy savings [27]. Fourth, reciprocity messaging—the desire to help people who have helped you—is another approach that has been shown to be effective for persuasion [28]. Fifth, moral messaging—appealing to an individual’s moral foundations (e.g. harm/care, authority) can convince people to change their mind on polarized issues, such as climate change, universal healthcare, military spending, and other politically-charged topics [29, 30]. This may work in the context of the COVID-19 pandemic given the politicization of the virus, but past work has tested the effect of moral reframing on support for policies and candidates, not public health behaviors. Researchers have also begun to analyze which types of persuasive messaging have been more effective within the context of the COVID-19 pandemic [24, 31–33]. However, we do not know the relative impact of all of these messages, because the studies have been conducted using different outcomes, different contexts, and different sample populations.

### The present research

The studies that follow had three main objectives. Our first objective was to systematically identify messages with high potential for persuasiveness from a large set of possible messages in the early stages of the pandemic. In two pre-tests (total *n* = 1,596), we tested how persuasive participants found 31 messages based on past research and 25 messages drawn from 600 crowdsourced messages, for a total of 56 messages.

Our second objective was to test whether reading a single persuasive message would impact intentions to engage in COVID-19 preventative behaviors, compared to a null control (i.e., seeing no message). In Study 1, which was pre-registered, we examined whether the four top-rated messages from the pre-tests and a standard public health message increased intentions to engage in preventive behaviors. We tested the messages in a between-subjects format, using the difference between people’s reported past behaviors before seeing a message and intended future behaviors after seeing a message as the dependent variable.

Our third objective was to test whether specific persuasive strategies can outperform a standard public health message with a description of the virus and recommended prevention behaviors. Using a standard message as an “active control”, we isolated the effects of a particular messaging approach, independent of the effect of receiving a reminder of the virus and desirable responses. In Studies 2 and 3, which were pre-registered, we tested whether any of the top-performing persuasive messages from the pre-tests could out-perform a standard public health message.

## Pretests

Our goal for the pretests was to identify promising messages to test further. All studies were approved by the Stanford University Institutional Review Board, and we obtained informed consent from all research participants.

### Method

In the first pretest of 598 participants, fielded in March of 2020, we measured perceptions of the persuasiveness of 24 short messages aimed at convincing people to stay home to prevent the spread of COVID-19. In the second pre-test of 998 participants, we used this same method, but added 7 additional messages from literature and 25 messages crowdsourced from participants in the first pre-test. We recruited participants from Mechanical Turk and Lucid (See S1 Appendix for more details about participants and recruitment). In the second pre-test, we also only included participants who indicated that they were not highly compliant with COVID behaviors, in order to find out which messages were most persuasive to those who most needed to change their behavior (see S1 Appendix).

The messages were based on a wide range of both general and prior research on persuasion and messaging, and research specific to the current pandemic. All messages were two to four sentences long and advocated for staying home to prevent the spread of coronavirus. See S1 Appendix for message text. The control message stated, “Coronavirus is a respiratory illness that can spread from person to person. The virus is thought to spread mainly between people who are in close contact with one another. You can help prevent the spread of COVID-19. Stay home and avoid contact with others when you must go out.” Each participant rated 10 messages for persuasiveness. The rating was a composite of three items measuring perceived persuasiveness (e.g. “How convincing do you find this reason for social distancing (staying home during the coronavirus pandemic)?”) on a scale from 1 to 9.

### Results

**Fig 1:**
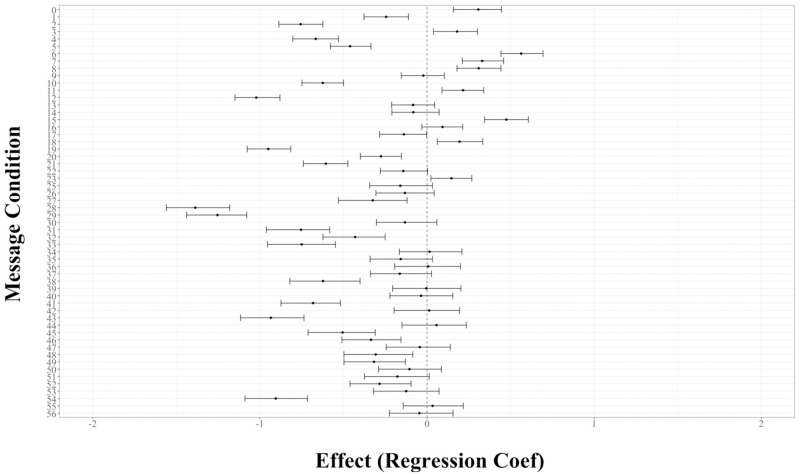
Effect size of messages relative to control in merged analysis of Studies 1 and 2 (95% confidence intervals). See S1 Appendix for message number key.

We used a mixed-effects model to calculate the main effects of message condition on persuasiveness, with a control message as the reference. The Level 1 effect was the message, and the Level 2 effect was the participant. We included controls for gender, age, race, education, and income. The outcome variable was perceived persuasiveness, which was the coefficient value associated with each message, non-standardized. (See S1 Appendix for full equation). We used the Holm method to adjust p-values to account for multiple comparisons. In a merged analysis of the two pre-tests, the five most persuasive messages emphasized responsibility to reciprocate sacrifices of healthcare workers (Message #6, *β*_6_ = 0.54, *p*_*holm*_ < .001), risk of overwhelming healthcare resources (Message #15, *β*_15_ = 0.46, *p*_*holm*_ < .001), the story of a sympathetic victim (Message #7, *β*_7_ = .32, *p*_*holm*_ < .001), protecting the vulnerable (Message #0, *β*_0_ = .29, *p*_*holm*_ < .001), and the speed of transmission (Message #8, *β*_8_ = 0.29, *p*_*holm*_ < .001). (Table 1) See Fig 1 for effect sizes of all messages when we pooled the data and included a dummy to indicate the first or second pre-test.

**Table 1:** Messages that outperformed control in merged analysis of Studies 1 and 2.

Message Number	Text	Effect size, Pre-test 1	Effect size, Pre-test 2	Effect size, Merged Analysis
6: Reciprocity	Doctors, nurses, and other health care workers are working around the clock, risking their lives to care for patients with the coronavirus. Working long hours in highly infectious environments, many of them are falling ill. As our health care workers put their lives on the line, we can do our part simply by staying home and limiting physical contact with others	0.60***	0.39**	0.54***
		(0.11)	(0.094)	(0.067)
15: Resource scarcity	Public health officials tell us that we must slow the spread of the coronavirus so numbers of sick people don’t overwhelm our doctors, nurses, and hospitals. If we don’t slow the spread, cases will increase rapidly, suddenly spiking beyond what the health care system can handle. We all can do our part to slow the spread by staying inside and avoiding contact with others when we must go out. If we take action to slow the spread now, we will save lives.	0.59***	0.21	0.46***
		(0.11)	(0.094)	(0.067)
7: Sympathetic victim	A few weeks ago, Fiona was a healthy 26-year-old with no medical complications. Then she suddenly came down with a bad cough and a feeling like she could not breathe. She tested positive for COVID-19, and is now hospitalized, receiving oxygen from a ventilator, and fighting for her life. This could be any of us. Please stay home and protect yourself against this virus!	0.35*	0.19	0.32***
		(0.11)	(0.095)	(0.067)
0: Protecting vulnerable	The sick, elderly, and immuno-compromised need our help. We all have a choice: If we go out, we risk the lives of others. But by staying home we can protect those most likely to be harmed. Stay home to protect those who are vulnerable!	0.34*	0.17	0.29 ***
		(0.11)	(0.095)	(0.066)
8: Speed of transmission	On average, each person passes on the coronavirus to 2 to 3 people, who then pass it on to more people, and so on. If you break a chain of transmission, you can single-handedly save lives and prevent the suffering of potentially dozens of people. Stay home as much as you can, and break the transmission chain!	0.48***	-0.02	0.29***
		(0.11)	(0.092)	(0.067)

Beta values are non-standardized.

All reported p-values are adjusted using the Holm correction for multiple hypothesis testing.

* p < .05;

** p < .01;

*** p < .001

## Study 1

In this study, we tested whether the four messages that were rated as most persuasive in the pre-tests would lead people who are not fully compliant to change their behaviors, compared to both a null control (no message) and a standard public health message.

### Method

#### Design and participants

This was a pre-registered, 6-condition, between-subjects study, with 4 treatment messages compared to a null control (no message) and an “active control” message that has a reminder of the virus and recommended behaviors. The active control message was the same as the control message in the pre-tests. We used both a null control and an active control in order to distinguish the effect of a specific message from the effect of reminding people of recommended behaviors. The four treatment messages were the messages that were significantly more persuasive than the control in the merged analysis of both pre-tests, and more persuasive than the control in the second pre-test, which focused on participants which were lower in compliance with public health behaviors (Messages #6, #7, #15, and #0). We made small edits to the messages to bring them up to date with current public health guidelines.

We used G*Power to calculate that 250 participants per condition would allow us to detect an effect size of Cohen’s d = 0.3 (Power = 80%, alpha = 0.05). Therefore, we targeted a sample size of 1500.

The study was fielded between May 22–23, 2020 using Lucid Marketplace. 5,180 participants began the survey. Based on our pre-registered exclusion criteria, we excluded participants who failed either an attention check at the start of the survey or one towards the end of the survey. After excluding participants who failed the first attention check, 4,149 remained (See S1 Appendix for attention check item). Only low and mid-level compliers with public health guidelines qualified to complete the survey. That is, we included people who reported engaging in at least two of the six behaviors: washed hands fewer than 10 times per day, cleaned surfaces fewer than 3 times a day, left the house for non-essential reasons at least once in past week, and marked less than 50 on a scale of 1 (Never) - 100 (Extremely often) for how frequently they kept six feet away from others, wore a face mask, and avoided touching their face when they left their home.

We also excluded participants who failed the second simple attention check, which was at the end of the survey. There was no differential attrition by condition based on this exclusion. After excluding participants for low attention and high COVID compliance, this left 1,627 participants. (48% male, median age = 47, 42% conservative, 30% moderate, 28% liberal). For the analyses that did *not* include the null control message, we also excluded participants who spent less than two seconds reading the message to account for inattentive participants, again following our pre-registered exclusion criteria. 1,072 participants met the inclusion criterion across the five conditions that were not the null control.

#### Procedure

*Change in intended behaviors*. After reading the message, participants indicated how often, in the next week, they intended to engage in the same six behaviors that they were asked about earlier (staying at home, wearing a mask, etc.). The items were on the same scale as the behavioral questions participants answered before reading the message, but were about intended future behaviors instead of past behaviors (see S1 Appendix). We took the difference between participants’ intended behaviors and past behaviors on each of the six items and normalized each one to be from 0–1. The main dependent variable was the unweighted average of these six normalized difference scores. We chose this variable because we wanted to measure whether the message caused people to take more precautionary behaviors in the future than they did in the past.

### Results

We ran two multiple linear regressions with the average normalized difference score across the six behaviors as the outcome variable. In the first model, the null control was the reference group. The additional covariates included in the model were 1) normalized measures of age, income, and 2) dummy variables for gender, education level and race/ethnicity. Compared to the null control, three messages and the active control message caused an increase in intentions to comply with public health guidelines, and the one message that was not significant showed trending effects (Table 2).

**Table 2:** Regression from Study 1, aggregate difference score as outcome.

Variable	Model 1	Model 2
	Null control as reference group	Active control as reference group
Active control	0.106*	--
	(.046)	
Message 6: Reciprocity towards healthcare workers	0.081.	0.0098
	(.046)	(.052)
Message 7: Sympathetic victim	0.165***	0.080
	(.045)	(.050)
Message 15: Resource scarcity	0.159***	0.0695
	(.046)	(.053)
Message 0: Protecting the vulnerable	0.159***	0.0337
	(.047)	(.053)
Normalized Age	-0.0159	-0.024
	(.014)	(.017)
Male	-0.142***	-.125***
	(.027)	(.033)
Education: HS or less	0.0783*	0.091.
	(0.038)	(.048)
Education: Some college	0.033	0.028
	(.034)	(.042)
Education: Postgraduate	-.004	-0.025
	(.045)	(.055)
Race: Asian	0.077	0.079
	(.060)	(.076)
Race: Black	0.212***	0.292***
	(.047)	(.063)
Race: Hispanic	0.125**	0.159*
	(.058)	(.080)
Race: Other	0.142	0.152
	(.09)	(.10)
Income	-4.0e-08	9.2e-08
	(2.8e-07)	(3.52e-07)
**Adjusted R-squared**	0.042	0.041
**Number of observations**	1627	1072

. p < .1;

* p < .05;

** p < .01;

*** p < .001

Standard errors are shown in parentheses.

Next, we conducted our pre-registered analysis. This was the same model, but excluding the null control and instead using the active control as the reference group. None of the four messages caused a significantly greater increase in preventative behaviors intentions than the active control (Table 2).

### Discussion

Participants who read any message advocating for COVID-19 preventive behaviors, including the active control message, reported significantly greater increases in intentions to engage in preventive behaviors than those in the null control condition. This suggests that seeing a reminder of the pandemic and recommended behaviors had an effect. However, no messages were significantly more persuasive than the active control message. This implies that the persuasive strategies had minimal effects. One possible reason for this is that we conducted the study at a time when many states were relaxing shelter-in-place orders for the first time since the start of the pandemic [34], and instead advocating for additional behaviors like wearing face masks. The messages may have seemed contradictory to “reopening” messaging prevalent at the time. In addition, Message #6 may not have seemed accurate—several participants expressed suspicion regarding whether healthcare systems were heavily impacted at that time.

## Study 2

We tested the persuasive messages again in Study 2. As noted above, one concern from Study 1 was that the behaviors the message called for may have been outdated. Strict stay-at-home orders were being lifted, and most public health officials were advocating for new guidelines, such as wearing face masks. Thus, in Study 2, we kept the same messaging, but modified the recommended behaviors.

### Method

#### Design and participants

Study 2 was a pre-registered, 5-condition study, comparing four treatment messages to an active control message. We adapted the same four messages from Study 1 to call for three new behaviors instead of asking people to stay home: physically distancing from others, wearing a mask, and washing hands after returning home (see S1 Appendix for message texts).

We fielded the study on Mechanical Turk from June 29 –July 1, 2020. At this point, the number of new COVID-19 cases per day had increased beyond the prior peak in April. Using G*Power, we found that a sample of 1,530 participants would allow us to detect a small effect size of Cohen’s d = 0.25 (Power = 80%, alpha = 0.05). We calibrated to a smaller effect size than in Study 1 given that there were no significant differences between messages in Study 1.

We excluded participants who failed the same attention checks as in Study 1 –the two simple attention checks as well as those who spent less than 2 seconds reading the message. We also excluded participants who were highly compliant with the three behaviors in the message. Participants reported how often they engaged in the three behaviors when they last left their place of residence, on a scale from 0 (*Never*) to 100 (*Extremely often*). We allowed participants to enter the study whose average on these three items was less than or equal to 93 out of 100. We chose this criterion because based on pilot data suggesting 50–60% of respondents would qualify. We slackened these criteria for two reasons. First, we did not see large differences in which messages were persuasive to the full sample in the first pretest and the low-compilers in the second pretest. Second, it would be beneficial to have 100% compliance on these behaviors, since participants can comply with them under almost all circumstances. Anyone who is not highly compliant has room for improvement.

3,058 unique respondents began the survey. 1,666 passed the first simple attention check and the filter for low-compliers. Of those, 1,531 passed the end attention check, and 1,421 participants spent at least two seconds on the page with the messages (58% male, median age = 34, 43% conservative, 19% moderate, 37% liberal). Note that this average age is much lower than the average age in Study 2, which reflects recruitment from different survey platforms.

#### Procedure

The procedure was similar to that of Study 1, but with new items before the messages to measure respondents’ skepticism toward COVID-19. To measure *skepticism of COVID*, participants answered two items on scales ranging from 0 (*Strongly disagree*) to 100 (*Strongly agree*) measuring how skeptical people were about the severity of COVID-19 (e.g. “Most people are overreacting to COVID-19.”) (*α* = 0.93). To measure *change in intended behaviors*, after reading the messages, participants indicated how often they intended to engage in the three behaviors listed in the message the next time they left their place of residence. We calculated the difference between the intended actions and reported past actions for each activity, and the main dependent variable was the average of these three differences (Mean = 5.0, Median = 7.0).

### Results

Consistent with our pre-registration, we ran the same regression model as in Study 1 to identify which messages caused a greater increase in intentions to engage in preventive behaviors. The most persuasive message was the reciprocity message (Message #6) (*β* = 2.47, p = .02), which was significantly more persuasive than the active control. No other messages were more persuasive than the active control (Table 3).

**Table 3:** Regression from Study 2 and 3, difference score (scale from 0–100) as outcome.

Variable	Study 2	Study 3
Message 6	2.47*	-0.765
	(1.08)	(0.89)
Message 7	0.52	--
	(1.07)	
Message 15	1.76	--
	(1.08)	
Message 0	1.11	--
	(1.08)	
Normalized Age	-0.21	2.82
	(0.328)	(0.5)
Gender: Male	-1.44*	-2.64**
	(0.69)	(0.91)
Education: HS or less	2.7*	1.45
	(1.25)	(1.64)
Education: Some college	2.35**	1.00
	(0.85)	(1.15)
Education: Post-graduate	0.66	1.27
	(0.94)	(1.27)
Race: Asian	2.56*	1.41
	(1.25)	(1.57)
Race: Black	0.584	1.12
	(1.12)	(1.41)
Race: Hispanic	0.62	3.96
	(1.77)	(1.73)
Race: Other	-1.92	4.84
	(3.07)	(5.35)
Income	-6.24e-06	2.5e-6
	(7.99e-06)	(9.4e-6)
**Adjusted R-squared**	**0.01**	**0.003**
**Number of observations**	**1427**	**568**

* p < .05;

** p < .01,

*** p < .001

Standard errors are shown in parentheses.

Most of the effect of the reciprocity message on intended compliance was driven by individuals low in skepticism about COVID-19. Among participants with skepticism rating below 50, those who viewed the reciprocity message (Message #6) (*β* = 3.49, p = .03) and the message about resource scarcity (Message #15) (*β* = 3.08, p = 0.05) reported a greater increase in intentions to comply compared to the active control message. There was no significant effect of any message among participants with skepticism scores greater than 50.

### Discussion

The reciprocity message, which was most persuasive in the pre-tests, was significantly more persuasive than the control in Study 2. However, the same message was not significantly more persuasive than the null control in Study 1. There are several reasons why results may have differed in Studies 1 and 2. First, the messages encouraged behaviors that were more in line with public health recommendations, whereas Study 1 messages may have contradicted other information respondents were receiving. Second, cases were rising when Study 2 was fielded (26), so it may have seemed more realistic that hospitals could be overwhelmed and there was therefore a need to reciprocate the actions of healthcare workers.

## Study 3

Given the different effects in Studies 1 and 2, we conducted a partial replication of Study 2, featuring only the reciprocity message and the active control.

### Method

#### Design and participants

This pre-registered study used a two-condition, between-subjects design. Otherwise, the procedure was the same as Study 2. We expected a small effect size, so using G*Power, we found that 620 participants would allow us to detect an effect size of Cohen’s d = 0.2 (80% power, alpha = 0.05).

The study was fielded on Mechanical Turk from July 14–15, 2020, when the number of new COVID-19 cases reported per day was increasing rapidly (25). Of the 1,212 participants who began the survey, 648 passed the behavior filter and simple attention item, 597 responded correctly to the end attention check, and 568 spent at least 2 second reading the message (52% male, median age = 34, 46% conservative, 18% moderate, 36% liberal).

#### Results

Using the same multiple linear regression as Study 2, Message #6 was not significantly more persuasive than the control (Table 3) in a two-tailed test. Though we conducted the study under nearly identical conditions approximately two weeks later, we did not find a significant effect of Message #6 on increase in intentions to comply.

### Discussion

The null effect may be because the earlier evidence for the reciprocity message were false positives. Alternatively, other changes in the world–e.g., crystallization of Americans’ views of the pandemic–may have led this message to no longer increase intentions to engage in preventive behaviors.

## General discussion

Across two within-subjects pre-tests where participants rated the persuasiveness of many short messages, several messages were significantly more persuasive than a control message. We then tested four messages in a between-subjects study, and all four led to an increase in intentions to comply with public health guidelines, compared to seeing no message. This suggests that seeing any of several different types of reminders of the virus and behaviors can increase intentions to engage in prevention behaviors. However, across three studies, none of the four messages consistently led to an increase in intentions to comply with public health guidelines compared to a standard public health message. We find that changes in the persuasive approach from short messages do not significantly affect participants’ intentions to increase compliance with public health guidelines. A message which emphasized civic responsibility to reciprocate sacrifices made by healthcare workers was rated most persuasive in the pre-tests out of 24 and 56 messages, respectively. It also showed a significant effect in Study 2. Yet it did not show an effect in a pre-registered replication.

There are several possible reasons why no messages were consistently more persuasive than a standard public health message. Coronavirus was in the news constantly and impacted almost every American. Small differences between short messages may not be persuasive in such a saturated information environment. It could be that there was a “persuasion window” early on when short messages could change behaviors, but the messages were no longer effective later on due to the overwhelming volume of messaging and the crystallization of views.

These null effects are consistent with other research on COVID-19 messaging. One study tested seven different messages designed to reflect moral values and relate these values to COVID-19. None of these messages significantly affected attitudes, intentions, or behaviors compared to a control condition [32]. Other work found that within a month of the COVID-19 shutdowns, prosocial messages were no longer effective at changing intended behaviors [24]. Beyond public health, research on the effectiveness of political ads, another salient issue, has also found that political ads for Presidential candidates and other salient elections have minimal effects [14, 15, 32, 35], and an experimental prime affected support for Trump’s candidacy in early 2016 but then had null effects later in the year [13]. Our results affirm prior research that it is unlikely any single, short, message would persuade people to change their behavior during the COVID-19 pandemic.

Our exploratory analysis in Study 2 is are broadly consistent with other work on COVID-19 messaging by showing that skepticism may be a moderator. In Study 2, we found that highly to moderately skeptical individuals were less likely to change their behavior in response to the short messages than individuals who were less skeptical. This is consistent with work [36] which found that people tend to think that individuals with slightly negative attitudes towards a behavior will be most persuadable, but actually, people with slightly positive attitudes towards a behavior are more persuadable.

In addition, we found that many messages in the pretests were rated as significantly *less* persuasive than a control message in our pre-tests. Messages evoking national identity, collective action, patriotism, analogies to war, religion, and purity were not perceived as highly persuasive. Likewise, messages highlighting vivid costs of not following guidelines (e.g., killing others, citations by law enforcement) were generally perceived to be less persuasive than the control. It may be that these messages read as direct persuasive efforts for certain subgroups, and prior work on direct vs. peripheral appeals suggest that some people often resist direct appeals [37]. Or, it might be that these messages are perceived as less authoritative. This finding is consistent with research [38] which found that messaging that were similar to the types of messages participants in the study expected from a health provider (not surprising, casual, or interactive) tended to be more effective at encouraging vaccination than surprising ones. This finding is also consistent with research on the “formality effect” [39], which found that formal messaging from government bodies was more effective than informal messaging for influencing behavior.

### Limitations

These experiments were conservative tests of messaging effects because their brevity may have led to minimal impact on participants. Participants read a very short message, without a clearly identified source, one time. The messages might be more persuasive if they were longer, delivered multiple times, or were conveyed via a more compelling medium, such as video. Similarly, one-on-one conversations [40] or receiving the message from a trusted source [41, 42] might have been more persuasive than decontextualized messages. It is possible that some of the persuasive strategies from the messages are effective, but only if delivered differently.

Another limitation is that we relied on self-reported past and intended future behaviors. Despite our care to include active controls, our dependent variable may still be susceptible to social desirability bias. In addition, the pandemic affected different regions of the country at different points in time, and health guidelines varied substantially by region. Some messages may have been effective in certain regions at certain times, but our studies were insufficiently powered to analyze regional differences.

### Future areas of research

This work points towards several possible areas of future research. First, more work could study whether there is a “persuasion window” after a crisis. There may be a short period of time after the start of a crisis, and before opinions crystallize, where messaging is effective at changing behavior. Second, more work could study heterogeneous treatment effects to find out which groups are most impacted by short messages. We find evidence suggesting that skepticism could moderate the relationship between messages and intended behavior change, and further research could study how baseline skepticism affects receptiveness to messages. Third, further work could study the effect of receiving multiple messages, longer messages, or more vivid messages. These may be more impactful than one-time, short messages and therefore be better tests. Fourth, future work could use adaptive experimental designs to efficiently test a larger number of messages.

## Conclusion

Across two pre-tests and three experiments conducted throughout five months of the coronavirus pandemic, results on the persuasiveness of different messaging strategies were inconsistent. We found that seeing a message with any reminder of the virus and prevention behaviors outperformed seeing no message at all. However, across three studies, none of the top-rated messages consistently outperformed a standard public health message. We generally found that the highest performing message highlighted reciprocity towards healthcare workers, but this effect did not replicate in the final study. Messages that are more frequent, more vivid, or from more credible sources may be more effective at increasing intentions to engage in prevention behaviors. These findings suggest that small changes in messaging are largely incapable of affecting intended compliance, even during early stages of the pandemic.

## Experimental protocols

All experimental protocols were approved by the Stanford University Internal Review Board and were carried out in accordance with relevant guidelines and regulations. Informed consent was obtained from all participants, and all participants were over the age of 18.

## Supporting information

S1 Appendix Additional methods and results. (PDF)
