# Supplementary material for: The effects of short messages encouraging prevention behaviors early in the COVID-19 pandemic
Source: PLoS One. 2023 Apr 14;18(4):e0284354. doi: 10.1371/journal.pone.0284354 (PMC10104278; doi:10.1371/journal.pone.0284354)
Supplement: S1 Appendix — (PDF) [file pone.0284354.s001.pdf]

S1 Appendix: Supporting Information

Pretests 1 and 2: Methods and Results **1**

List of Messages **9**

Results for All Pretest Messages **20**

Compliance Filter Items, Dependent Variables, and Attention Check Questions **33**

Message Text for Studies 2 and 3 **36**

Merged Analysis of Studies 2 and 3 **37**

Pretests 1 and 2: Methods and Results

Pre-test 1

Method

Participants

Prior to the study, we conducted power analyses using G*Power (Faul et al., 2007), which showed 200 participants per pairwise comparison would be needed to detect an effect of Cohen's d = 0.3 (95% power, alpha = 0.01). With 24 treatment messages, and with each participant rating 10 messages, we aimed for a total sample size of at least 480 (200 views per message * 24 messages / 10 messages per person). The final sample was N = 598 (275 men, 323 women, Mean age = 43.8, 42% liberal, 14% moderate, 44% conservative). Participants on Mechanical Turk (a crowdsourcing marketplace commonly used for academic studies) completed the survey from March 27-30, 2020. At this point in the pandemic, some parts of the US were already under stay-at-home orders (Kates, 2020).

Procedure

We tested 24 short messages and a control message. The messages were based on a wide range of both general and prior research on persuasion and messaging, and research specific to the current pandemic. Although the messaging strategies are not all-inclusive, we aimed to collect a diverse set of strategies from past literature. All messages were two to four sentences long and advocated for staying home to prevent the spread of coronavirus. The messages were not accompanied by any graphics. Examples of persuasive techniques and quotes from the corresponding messages are below.

- *Social proof* (Schultz et al., 2007): "90% of Americans are significantly changing their behavior"
- *Reciprocity* (Cialdini et al., 1992): "As our health care workers put their lives on the line, we can do our part simply by staying home."
- *Formal authority* (Cialdini, Ch. 6, 1987): "in many parts of the US, you can be cited for a misdemeanor"
- *Moral purity* (Feinberg & Willer, 2013): "effects [of COVID-19] are disgusting, threatening our bodies by damaging tissue and impairing our breathing."
- *Patriotism* (Feinberg & Willer, 2015): "we all have a patriotic duty to do whatever we can to help our country."
- *Compassion and empathy for others* (Uskul & Oyserman, 2010): "The sick, elderly, and immunocompromised need our help."
- *Deontological and utilitarian morality* (Everett et al., 2020): "it's your duty" and "think of the consequences," respectively.

- *Emphasizing personal health threat* (Dunlop et al., 2009): "Young, healthy people all over the country are being hospitalized [...] This virus can hurt every one of us."
- *Explaining dynamics of viral spread* (Lammers et al., 2020): "on average, each person passed on the coronavirus to 2 to 3 people"
- *Political partisanship* (Cohen, 2003): "President Trump and the Coronavirus Task Force have ordered all Americans…"
- *Identifiable victim* (Small & Lowenstein, 2005) : "Fiona was a healthy 26-year-old [...] and is now hospitalized, receiving oxygen from a ventilator, and fighting for her life."

Each participant viewed ten messages randomly selected from the set of 24 possible messages in a random order. Each message was rated 236 to 242 total times.

The control message stated, "Coronavirus is a respiratory illness that can spread from person to person. The virus is thought to spread mainly between people who are in close contact with one another. You can help prevent the spread of COVID-19. Stay home and avoid contact with others when you must go out." This message was based on language from the CDC's website and deliberately does not use a persuasive method beyond stating facts that are common to most public health messaging about COVID-19 at the time of the study.

Persuasiveness: After reading each message, participants answered three questions gauging how persuasive they found the message: "How convincing do you find this reason for social distancing (staying home during the coronavirus pandemic)?" on scale from 1 (*Not convincing at all)* to 9 (*Extremely convincing),* "After reading this message, how likely are you to stay home and avoid social contact for at least the next two weeks?" on scale from 1 *(Extremely unlikely)* to 9 *(Extremely likely),* and "Would you share this message on social

media?" on a scale from 1 (*Definitely would not share*) to 9 (*Definitely would share*). Reliability was high, (α = .80) so we combined these into a composite.

Crowdsourced Messages: At the end, we asked participants to write their own two to four sentence message that they thought "might convince someone to follow current CDC guidelines like maintaining social distance and staying home except for essential reasons."

Results

We used a mixed-effects model to calculate main effects of message condition on persuasiveness, with a control message as the reference. The level 1 effect was the message, and the level 2 effect was the participant. We included controls for gender, age, race, education, and income. The equation is below:

DV(ij) = b00 + b10*MessageCondition(ij) + b20*OrderCondition(ij) + b01*PolitFactor(j) + b02*Gender(j) + b03*Age(j) + b04*Race(j) + b05*Education(j) + u0(j) + r(ij)

The outcome variable was the beta value associated with each message, non-standardized. We used the Holm method to adjust p-values to account for multiple comparisons. The five most persuasive messages emphasized responsibility to reciprocate sacrifices of healthcare workers (Message #6, β = 0.60, $p_{holm} < .001$), importance of following guidance of public health officials (Message #15, β = 0.59, $p_{holm} < .001$), the speed of transmission (Message #8, β = 0.48, $p_{holm} < .001$), the story of an identifiable victim (Message #7, β = .35, $p_{holm} = .02$), and protecting the vulnerable (Message #0, β = .34, $p_{holm} = .02$). (See Table S1.)

Many messages were rated as less persuasive than the control, including messages emphasizing: political party by referencing President Trump (Message #19, β = -1.33, $p_{holm} < .001$), religion (Message #12, β = -1.3 $p_{holm} < .001$), and patriotism (Message #5, β = -0.62 $p_{holm} < .001$). Interestingly, messages that emphasized these typically conservative values were also less persuasive than the control among conservatives.

One limitation is that at the time this study was fielded, most Americans were already complying with social distancing guidelines (Murad, 2020). It is possible that people who were already complying might find different messages convincing than people who are not, and the latter population is of greatest interest.

Pre-test 2

Pretest 2 relied on the same protocol as Pretest 1, with two exceptions. First, only participants who were not highly compliant with public health behaviors were included. Second, we added 7 messages from related studies and 25 crowdsourced messages.

Method

Participants

A total of 998 participants from Lucid (851 participants) and Mechanical Turk (147 participants) were included in our final analysis. The survey was in the field from April 16 - May 1, 2020. At this point in time, the number of new COVID-19 cases per day in the US was plateauing (CDC, 2020). We expanded the sample platform to Lucid, another survey provider, because of reports that participants on Mechanical Turk were seeing a large number of studies related to COVID-19, and we had concerns about oversaturation. Using G*Power, we found that 150 participants per pairwise comparison between a treatment message and a control message would be needed to detect an effect of Cohen's d = 0.3 (95% power, alpha = 0.05). Because each

participant would see 9 treatment messages and the control, our target sample was 933 (150 views per message * 56 messages / 9 view per participant = 933 participants).

We only allowed participants to enter the study who passed a simple attention check (see Supplementary Materials). Of the 7,658 participants who began the survey, 6,268 passed this check. We also sought to include only participants who were low in compliance with public health guidelines. Before allowing participants to take the full survey, participants indicated how often they engaged in six behaviors that were widely recommended to prevent the spread of coronavirus. We aimed to identify the approximately 20% of the population lowest in compliance. A brief pilot test led us to only allow participants that met at least five of the following six criteria: washed hands fewer than 10 times per day, cleaned surfaces fewer than 3 times a day, left the house for non-essential reasons at least once in past week, and marked less than 50 on a scale of 1 (*Never*) - 100 (*Extremely often*) for how frequently they kept six feet away from others, wore a face mask, and avoided touching their face when they left their home..

Among those who began the study, this filter excluded all but 998 participants (54% male; median age = 42; 55% conservative, 20% moderate, 25% liberal).

New Messages

We added 7 new messages from in-progress research on COVID-19 and persuasive techniques we saw in the field that we did not include in Study 1. These include:

- *Conflict mindset* (Kleiman & Enisman, 2018): “We are in a once-in-a-lifetime fight against a merciless enemy.”
- *Dynamics of transmission:* (Luttrell, 2020): “Social distancing helps prevent you from unknowingly transmitting the virus”

Crowdsourced Messages

In Study 1, we collected 600 messages written by participants. Two research assistants indicated the persuasiveness of each message on a scale of 1 (*Not convincing at all*) to 7 (*Extremely convincing*), and distinctiveness from the 24 messages tested in Study 1 on a scale of 1 (*Almost identical*) to 7 (*Extremely distinct*). To identify potentially persuasive messages that were distinct from Study 1 messages, we calculated the weighted average of these two items ($0.4 * Rating_{persuasiveness} + 0.6 * Rating_{distinctiveness}$), and included the 25 messages with the highest average score as treatment messages in Pretest 2. Distinct messaging strategies included emphasizing personal responsibility for harm – e.g. "your actions may cause people to get sick and die" – and explaining how preventative behaviors would speed recovery and help us "get back to normal" (See Supplementary Materials.)

Procedure

The procedure was modified only slightly from Pretest 1. Here, all participants saw the control message and nine randomly selected treatment messages, instead of 10 randomly selected messages. Messages were shown in a random order. Each treatment message was seen 148 to 173 times.

Results

The most persuasive message was again Message #6, which emphasized a civic obligation to protect healthcare workers (β = 0.39, p_{holm} = .01). The second most persuasive message overall was Message #15, which emphasized the need to follow guidelines of public health officials. However, this effect was insignificant when using the Holm adjustment for p-values (β = 0.21, $p_{unadjusted}$ = .03, p_{holm} = .9). (Table 1)

None of the crowdsourced messages were significantly more persuasive than the control message. Many messages were significantly less persuasive than the control, including messages

that place the COVID-19 pandemic as the "challenge of our times" (Message #33, β = -0.74, $p_{holm} < .001$) or as something that "historians will look back on" (Message #38, β = -0.60, $p_{holm} < .001$).

We also conducted a combined analysis, merging data from Pretests 1 and 2, and adding a dummy variable to indicate study.

Discussion of Pretests

Top messages in Pretests 1 and 2 were similar. However, in Pretest 2, the effect size of the most persuasive messages was much smaller, likely due the shift to sampling a low-compliance population. In both studies, the most persuasive message emphasized civic responsibility to reciprocate healthcare workers' sacrifices. Overall, the more persuasive messages emphasized tended to emphasize how staying home protects others.

The more persuasive messages emphasized positive effects of staying home, such as "slowing the spread" or "saving lives." Less persuasive messages highlighted negative consequences, such as being "cited for a misdemeanor," or "killing" others. This is consistent with findings that gain-framed messages are more effective than loss-framed messages for encouraging preventative behaviors (O'Keefe & Jensen, 2006).

We tested for differences between liberals and conservatives, as some of the messages used typically conservative values, and some used typically liberal values. (See Supplementary Materials). We found that similar messages were most persuasive across all ideologies – for example, Messages #6 and #15 were among the top three most persuasive messages among liberals, conservatives, and moderates. Appeals to traditionally conservative values, such as religion (Message #12), purity (Message #2), or the Republican party (Message #19) were rated

as unconvincing, even to conservatives. One difference that the most persuasive messages had a larger effect size compared to the active control among liberals than among conservatives.

The majority of the messages in Pretest 2 were less persuasive than the control. This is likely in part due to the low-compliance population being more skeptical about the messages, and in part due to crystallization of views over time. Messages that highlighted collective action, such as analogies to war (Message #4), the need to "stick together" (Message #10), or social proof (Message #20) were all rated as less persuasive than the control. This may be because in individualistic cultures, messages emphasizing collectivism are less persuasive (Sherman et al., 2011). Techniques from the crowdsourced messages, such as emphasizing personal responsibility for harm (Message #46) or the need to "get back to normal" (Message #53) also not significantly persuasive.

Overall, participants thought that the majority of messages were less persuasive or equally persuasive to a control. Although only five messages were more persuasive than the control in the merged analysis, 21 messages were significantly less persuasive. The results provide insights on which messages to avoid. A simple message stating the facts about COVID-19, without any specific persuasive strategy, can be more effective than many messages.

List of Messages

All message numbers, text, source of the message, and hypothesized mechanism are below. Messages 0-24 were used in Pretest 1. Messages 0-56 were used in Pretest 2. Messages 0, 6, 7, 15, and the control were used in Studies 1-3.

The control message for the Pretests and Study 1 was: *Coronavirus is a respiratory illness that can spread from person to person. The virus is thought to spread mainly between people who are in close contact with one another. You can help prevent the spread of COVID-19. Stay home and avoid contact with others when you must go out.*

Table S1: All Messages

Number	Message	Source	Mechanism	Citation
0	The sick, elderly, and immuno-compromised need our help. We all have a choice: If we go out, we risk the lives of others. But by staying home we can protect those most likely to be harmed. Stay home to protect those who are vulnerable!	Paper authors	Compassion and empathy	Stiff, J. B., Dillard, J. P., Somera, L., Kim, H., & Sleight, C. (1988). Empathy, communication, and prosocial behavior. Communication Monographs, 55(2), 198–213. https://doi.org/10.1080/03637758809376166
1	As the threat of coronavirus builds, we as a society must act, and act together. Our efforts to prevent the spread of the virus are only as strong as the weakest link. One or two among us who fail to take this threat seriously risk harming to us all. We must stay home!	Paper authors	Public good, collectivism	Uskul, A. K., & Oyserman, D. (2010). When message-frame fits salient cultural-frame, messages feel more persuasive. Psychology & Health, 25(3), 321–337. https://doi.org/10.1080/08870440902759156
2	Coronavirus is a deadly sickness. Its effects are disgusting, threatening our bodies by damaging tissue and impairing our breathing. If not stopped, this sickness will corrode our communities, infect our families, and spread beyond the control of our medical facilities. Stay home to protect yourself from this vile sickness!	Paper authors	Purity moral frame	Feinberg, M., & Willer, R. (2013). The moral roots of environmental attitudes. Psychological science, 24(1), 56-62.
3	It's not only the elderly who get seriously ill from coronavirus. Young, healthy people all over the country are being hospitalized and are fighting for their lives due to the coronavirus. This virus can hurt every one of us. Stay home to protect yourself!	Paper authors	Emphasis on harm	Feinberg, M., & Willer, R. (2019). Moral reframing: a technique for effective and persuasive communication across political divides. Social and Personality Psychology Compass, 13(12), e12501.
4	During times of war, Americans sacrifice to protect one another and support our military. Now, coronavirus has invaded our nation and we must take similar steps to protect ourselves and our fellow Americans. The most patriotic action you can take is to stay home as much as possible!	Paper authors	Patriotism moral frame	Feinberg, M., & Willer, R. (2019). Moral reframing: a technique for effective and persuasive communication across political divides. Social and Personality Psychology Compass, 13(12), e12501.
5	As Americans we all have a patriotic duty to do whatever we can to help our country. Our nation's public health officials tell us that the	Paper authors	Patriotism moral frame	Feinberg, M., & Willer, R. (2019). Moral reframing: a technique for

	best strategy now is to stay home to slow the spread of the coronavirus. Please join together with your fellow Americans. We all must do our part by staying home.			effective and persuasive communication across political divides. Social and Personality Psychology Compass, 13(12), e12501.
6	Doctors, nurses, and other health care workers are working around the clock, risking their lives to care for patients with the coronavirus. Working long hours in highly infectious environments, many of them are falling ill. As our health care workers put their lives on the line, we can do our part simply by staying home and limiting physical contact with others.	Paper authors	Principle of reciprocity	Cialdini, R. B., Green, B. L., & Rusch, A. J. (1992). When tactical pronouncements of change become real change: The case of reciprocal persuasion. *Journal of Personality and Social Psychology*, *63*(1), 30.
7	A few weeks ago, Fiona was a healthy 26-year-old with no medical complications. Then she suddenly came down with a bad cough and a feeling like she could not breathe. She tested positive for COVID-19, and is now hospitalized, receiving oxygen from a ventilator, and fighting for her life. This could be any of us. Please stay home and protect yourself against this virus!	Paper authors	Identifiable victim	Small, A., & Loewenstein, G. (2003). Helping a Victim or Helping the Victim: Altruism and Identifiability: Journal of Risk and Uncertainty
8	On average, each person passes on the coronavirus to 2 to 3 people, who then pass it on to more people, and so on. If you break a chain of transmission, you can single-handedly save lives and prevent the suffering of potentially dozens of people. Stay home as much as you can, and break the transmission chain!	Paper authors	Technical frame; correcting mispercepti ons about exponential growth	Voelkel, J. G., Mernyk, J., & Willer, R. (2020, May 17). Resolving the Progressive Paradox: The Effects of Moral Reframing on Support for Economically Progressive Candidates. https://doi.org/10.31234/osf.io/mtfjn,Lammers, J., Crusius, J., & Gast, A. (2020). Correcting misperceptions of exponential coronavirus growth increases support for social distancing. Proceedings of the National Academy of Sciences, 117(28), 16264-16266.
9	This pandemic is a critical moment in our history. We all will look back and ask ourselves what we did in this time. Public health officials tell us that the most important thing for us to do now is stay home as much as possible, and avoid close	Paper authors	Future self-continui ty	Hershfield, H. E. (2011). Future self-continuity: How conceptions of the future self transform intertemporal choice. Annals of the New York

	physical contact when we must go out. Please stay home to stop the spread of this deadly virus.			Academy of Sciences, 1235, 30.
10	Although times are tough right now for many Americans, we must stick together and take actions that benefit the greater good. We MUST stick together regardless of our ethnic, religious, or cultural backgrounds through these challenging times.	Paper authors	Collectivism	Uskul, A. K., & Oyserman, D. (2010). When message-frame fits salient cultural-frame, messages feel more persuasive. Psychology & Health, 25(3), 321–337. https://doi.org/10.1080/08870440902759156
11	Every single man, woman, and child is at great risk during the coronavirus pandemic. While older people and those with compromised immune systems are at greatest risk, middle aged people can also get sick and die from the virus. The overloading of the healthcare system puts us all at risk. We must act now to slow the spread of this virus.	Paper authors	Self-protection	Dunlop, S. M., Wakefield, M., & Kashima, Y. (2010). Pathways to Persuasion: Cognitive and Experiential Responses to Health-Promoting Mass Media Messages. Communication Research, 37(1), 133–164. https://doi.org/10.1177/0093650209351912
12	As people of faith, we must join together to slow the spread of the coronavirus. This pandemic is a test of our faith and our community. We must stand by our beliefs and, most importantly, do our part to protect others. By staying home and avoiding contact when we must go out, we can slow the spread of this virus.	Paper authors	Religion frame	Putnam, R. D., & Campbell, D. E. (2012). American grace: How religion divides and unites us. Simon and Schuster.
13	STAY HOME, even if you don't feel sick. Coronavirus is contagious even before you have symptoms. We all need to do this, however difficult, because it is the right thing to do: it is our duty to protect our families, friends, and fellow citizens. IT'S YOUR DUTY.	Everett et. al.	Deontological moral frame	Everett, J. A. C., Colombatto, C., Chituc, V., Brady, W. J., & Crockett, M. (2020, March 20). The effectiveness of moral messages on public health behavioral intentions during the COVID-19 pandemic. https://doi.org/10.31234/osf.io/9yqs8
14	STAY HOME, even if you don't feel sick. Coronavirus is contagious even before you have symptoms.We all need to do this, however difficult, because these sacrifices are nothing compared to the much worse consequences for everyone if we carry on as	Everett et. al.	Utilitarian moral frame	Everett, J. A. C., Colombatto, C., Chituc, V., Brady, W. J., & Crockett, M. (2020, March 20). The effectiveness of moral

	usual. THINK OF THE CONSEQUENCES.			messages on public health behavioral intentions during the COVID-19 pandemic. https://doi.org/10.31234/osf.io/9yqs8
15	Public health officials tell us that we must slow the spread of the coronavirus so numbers of sick people don't overwhelm our doctors, nurses, and hospitals. If we don't slow the spread, cases will increase rapidly, suddenly spiking beyond what the health care system can handle. We all can do our part to slow the spread by staying inside and avoiding contact with others when we must go out. If we take action to slow the spread now we will save lives.	Paper authors	Appeal to experts	Cialdini, R. B. (1987). Influence (Vol. 3). Port Harcourt: A. Michel.
16	It is critical for us all to follow the direction of our public health officials by staying inside and avoiding contact with others when we must go out. Some people have ignored these guidelines by going on spring break trips or hosting parties, with terrible consequences for those they come in contact with. Disregarding public health guidelines isn't just irresponsible. It's also selfish and wrong, because it puts others at risk of serious illness and even death.	Paper authors	Moral judgement of free-riders	Fehr, E., & Gachter, S. (2000). Cooperation and punishment in public goods experiments. American Economic Review, 90(4), 980-994.
17	The coronavirus pandemic is a great test of our toughness. To win in this time we must be firm and we must be brave. The most effective thing most of us can do right now is to STAY INSIDE and avoid contact with others when we must go out. Making these and other sacrifices is not easy. It requires courage and grit. But we do it for our friends and our neighbors. And they do the same for us.	Paper authors	Appeal to masculinity; in-group loyalty	Feinberg, M., & Willer, R. (2019). Moral reframing: a technique for effective and persuasive communication across political divides. Social and Personality Psychology Compass, 13(12), e12501.
18	Many people carry the coronavirus without displaying symptoms. This means that we should all think and act as though we MIGHT have the virus. The best thing you can do to protect yourself and your family is to take the same precautions you would take -- staying home, washing hands -- as you would if you had the virus.	Paper authors	Act like you have it	Common in messaging from the Prime Minister of New Zealand (https://www.reuters.com/article/us-health-coronavirus-newzealand-emergen/act-like-you-have-covid-19-pm-ardern-says-as-new-zealand-heads-into-lockdown-idUSKBN21C061), the US surgeon general (https://www.independent.co.uk/news/world/americ

				as/coronavirus-us-lockdown-how-many-cases-surgeon-general-a9409886.html) and other persuasive articles.
19	President Trump and the Coronavirus Task Force have ordered all Americans to avoid gatherings of 10 or more people and to stay home as much as possible. They are right! We should listen to the President: stay home!	Paper authors	Appeal to authority	Cohen, Jeoffrey L. 2003. "Party over Policy: The Dominating Impact of Group Influence on Political Beliefs." Journal of Personality and Social Psychology 85 (5): 808–22.
20	Over 90% of Americans are significantly changing their behavior to reduce the spread of coronavirus. Two-thirds of Americans are mostly or completely isolating themselves. Join most Americans in slowing the spread of the virus: stay home!	Paper authors	Principle of social proof	Schultz, P. W., Nolan, J. M., Cialdini, R. B., Goldstein, N. J., & Griskevicius, V. (2007). The constructive, destructive, and reconstructive power of social norms. *Psychological Science*, *18*(5), 429-434.
21	If you leave your home for non-essential activities in many parts of the US, you can be cited for a misdemeanor. Obey the law. Stay home.	Paper authors	Appeal to authority; threat of punishment	Cialdini, R. B. (1987). Influence (Vol. 3). Port Harcourt: A. Michel.
22	In the fight against coronavirus, every day matters. Staying at home and avoiding social contact just one day earlier can make a great difference in the spread of the virus and can save lives. Please start social distancing NOW.	Paper authors	Urgency effect	Zhu, M., Yang, Y., & Hsee, C. K. (2018). The mere urgency effect. Journal of Consumer Research, 45(3), 673-690.
23	Doctors, nurses, grocery store clerks, and others are risking their lives to keep us safe. To keep them as safe as possible, we must STAY HOME! It's our way of helping them do their jobs. Together, we can save lives and beat the virus.	Paper authors	Reciprocity	Cialdini, R. B. (1987). Influence (Vol. 3). Port Harcourt: A. Michel.
24	**Coronavirus is a respiratory illness that can spread from person to person. The virus is thought to spread mainly between people who are in close contact with one another. You can help prevent the spread of COVID-19. Stay home and avoid contact with others when you must go out.**		Control Message	
25	If we all practice social distancing together, the virus is less likely to spread to people	Andy Luttrell		Luttrell, A. (2020, July 15). Moral Matching and

	who are most at-risk (i.e., older adults and people with suppressed immune systems).			Social Distancing Arguments. Retrieved from osf.io/fwhva
26	If we all practice social distancing together, the virus will spread less quickly, which will reduce the burdens on our health care system.	Andy Luttrell		Luttrell, A. (2020, July 15). Moral Matching and Social Distancing Arguments. Retrieved from osf.io/fwhva
27	Practicing social distancing helps prevent you from unknowingly transmitting the virus to who may go on to transmit the virus to even more people.	Andy Luttrell		Luttrell, A. (2020, July 15). Moral Matching and Social Distancing Arguments. Retrieved from osf.io/fwhva
28	Practicing social distancing is what everybody else is doing.	Andy Luttrell	Social Proof	Luttrell, A. (2020, July 15). Moral Matching and Social Distancing Arguments. Retrieved from osf.io/fwhva
29	Practicing social distancing means complying with what government officials are demanding.	Andy Luttrell	Appeal to authority	Luttrell, A. (2020, July 15). Moral Matching and Social Distancing Arguments. Retrieved from osf.io/fwhva
30	We are in a once-in-a-lifetime fight against a merciless enemy - a virus that could kill millions of people. Public health officials tell us that the key to defeating this enemy is to stay inside and avoid contact with others, so that we can keep the virus isolated. If we all do our part to contain the virus, then we can win this fight together.	Dan Greene	Conflict Mindset	Kleiman, T., & Enisman, M. (2018). The conflict mindset: How internal conflicts affect self-regulation. Social and Personality Psychology Compass, 12(5), e12387. https://doi.org/10.1111/spc3.12387
31	And then the whole world walked inside and shut their doors and said: We will stop it all. EVERYTHING. To protect our weaker ones. Our sicker ones. Our older ones. And nothing. NOTHING in the history of humankind ever felt more like LOVE than this.	Facebook	Collectivism/public goods	
32	There is nothing more important than giving your time to another person. Right now, your time spent at home social distancing is a gift of time to millions and in return those millions are gifting time to you. Let's keep everyone's clocks ticking!	mTurk worker		
33	Covid-19 is the challenge of our times. Our great-grandparents fought WWII, our	mTurk worker		

	grandparents helped save the world from communism, our parents defeated segregation, it is time for us to rise to the occasion. Stay home!			
34	Our elderly population has spent their lives sacrificing and caring for us. Do your part to thank them by protecting them from the spread of the coronavirus by staying home as much as you can.	mTurk worker		
35	As the CDC and WHO say, you can be asymptomatic up to 14 DAYS! PLEASE stay home to avoid passing it to others whom it may KILL! YOU can save lives simply by staying home. Your grandparents went to war to save the world, all you have to do is stay on the couch to save the world!!	mTurk worker		
36	We've all experienced the false alarms of pandemics in the past by our questionable media. But this time the threat is real. Covid-19 is deadly and easily contracted. Anyone you interact with can be infected even if they are not showing symptoms. This means you can easily become infected and spread the disease to your loved ones. Don't play Russian roulette: The safest thing you can do for your family and loved ones is stay home and practice social distancing. The people you love are counting on you.	mTurk worker		
37	Our parents and grandparents were called to war to protect us, we're being asked to stay home now to protect them. Protect your loved ones, the health care workers, by staying home.	mTurk worker		
38	Historians are going to look at this time and study what people did to protect themselves and others. What will you tell your children you did? If it turns out that you have coronavirus and have to tell public health officials where you've been in the past two weeks, would you be proud or ashamed?	mTurk worker		
39	If someone gets the virus they are sent to the hospital alone. No family to support them. No family to sit with them. No family to say good bye if they don't make it. They are ALL ALONE no matter what their age is! ALONE! Stay home and take care of your family	mTurk worker		

40	My daughter-in-law is a nurse and she is working very long hours trying to help her patients survive after they have gotten infected from the Coronavirus. One of her patients is a grandfather who caught the virus from his granddaughter. Stay home and protect your own family by maintaining social distance from those who are at most risk for getting infected.	mTurk worker		
41	Be a hero! Each of us can be a hero in defeating this virus...just stay home. Keep your friends, neighbors and relatives virus free...just stay home. You can be a hero. Be a hero.	mTurk worker		
42	Do you have COVID-19? No? Are you sure? Some people show no symptoms. Are you okay with possibly being infected and going out and infecting others? The ones you love? How would you feel if your friend got sick because of you? Or your own mother? When you think about social distancing, don't just think about yourself. Think about all the important people in your life... and losing them.	mTurk worker		
43	Please don't kill my family - stay home and I will too so I don't kill yours.	mTurk worker		
44	You can do your part! We can help each other in these tough times, if we all do our part we can beat COVID-19. Stay home, we can protect those who are most vulnerable by staying home, don't wait until you are directly affected. Remember you can save someone's mom, grandfather, uncle. WE CAN DO IT!	mTurk worker		
45	Plenty of people have already made sacrifices to make this world the way it is. This is your time to pay it forward. Think of others because others are already thinking about you.	mTurk worker		
46	If you don't practice social distancing you may inadvertently end up killing a close friend or family member. How could you possibly live with that? Stay home	mTurk worker		
47	When you stay home, you're giving other's a fighting chance. You're giving grocery store workers time to restock the shelves and protect themselves. You're giving emergency health workers a chance to focus on	mTurk worker		

	individual patients. You're giving your grandma a chance to avoid the virus.			
48	Stopping the virus in its tracks will allow scientists working on vaccines/cures time to help as they can. Taking just one person out of the equation could help hundreds or thousands down the line. Stay home for the sake of us all.	mTurk worker		
49	The coronavirus, aka COVID-19, knows no borders and shows no mercy. The future survival of the human race depends on you doing the right thing by staying home and self-quarantining for 14 days. You can still interact with friends and loved ones on your preferred platforms and can stay in touch with them by calling, texting or videochatting. Self-quarantine does not mean no virtual contact.	mTurk worker		
50	I am a nurse. I have seen things no one should have to endure. Please listen and protect yourself and your loved ones by staying home.	mTurk worker		
51	Don't risk the health and well-being of your neighbors and loved ones. Unless it's of absolute necessity, stay home to prevent the spread of the coronavirus. Our grandparents risked their lives fighting over seas; you can save lives by sitting at home on your sofa.	mTurk worker		
52	Take responsibility for your own actions. Your actions may cause people to get sick and die.	mTurk worker		
53	This outbreak would be over a lot sooner if we just stayed inside. If people do not listen then this is just going to get worse and prolong everything. The sooner we can stop this the sooner we can return to our lives as normal	mTurk worker		
54	People should begin staying at home and following the recommendations of the CDC before we lose even more of the freedoms that we as Americans cherish. No one wants martial law.	mTurk worker		
55	We must continue to social distance and stay at home as much as possible. We are in absolute danger of having our hospitals and clinics overwhelmed and becoming infection sites for loved ones who are under medical	mTurk worker		

	care for cancer, stroke, or accidents. Please show you care, stay home.			
56	Expressing rebellion by breaking quarantine, going out for non-essentials, or socializing etc..., will not only spread the virus but will also accomplish nothing except longer shutdowns and extended quarantines for all of us. The virus does not move itself, it is moved by people. IF we stop moving so does the virus and life can get back to normal sooner.	mTurk worker		

Results for All Pretest Messages

The mixed-effects model we used was the following. The coefficient of interest is b10 in the equation, beta in the table.

Persuasiveness(ij) = b00 + b10*MessageCondition(ij) + b20*OrderCondition(ij) + b01*PolitFactor(j) + b02*Gender(j) + b03*Age(j) + b04*Race(j) + b05*Education(j) + b06*Income(j)+ u0(j) + r(ij)

Control message: "Coronavirus is a respiratory illness that can spread from person to person. The virus is thought to spread mainly between people who are in close contact with one another. You can help prevent the spread of COVID-19. Stay home and avoid contact with others when you must go out."

Table S2: Messages that were significantly more persuasive than the control in merged analysis.

#	Message Text	Source	Mechanism	Beta	P-value, unadjusted	P-value, Holm adjusted
6	Doctors, nurses, and other health care workers are working around the clock, risking their lives to care for patients with the coronavirus. Working long hours in highly infectious environments, many of them are falling ill. As our health care workers put their lives on the line, we can do our part simply by staying home and limiting physical contact with others.	paper authors	Principle of reciprocity	0.54	8.63E-16	3.97E-14
15	Public health officials tell us that we must slow the spread of the coronavirus so numbers of sick people don't overwhelm our doctors, nurses, and hospitals. If we don't slow the spread, cases will increase rapidly, suddenly spiking beyond what the health care system can handle. We all can do	paper authors	Appeal to experts	0.46	7.62E-12	3.12E-10

	our part to slow the spread by staying inside and avoiding contact with others when we must go out. If we take action to slow the spread now, we will save lives.					
7	A few weeks ago, Fiona was a healthy 26-year-old with no medical complications. Then she suddenly came down with a bad cough and a feeling like she could not breathe. She tested positive for COVID-19, and is now hospitalized, receiving oxygen from a ventilator, and fighting for her life. This could be any of us. Please stay home and protect yourself against this virus!	paper authors	Identifiable victim	0.31	2.89E-06	1.10E-04
8	On average, each person passes on the coronavirus to 2 to 3 people, who then pass it on to more people, and so on. If you break a chain of transmission, you can single-handedly save lives and prevent the suffering of potentially dozens of people. Stay home as much as you can, and break the transmission chain!	paper authors	Technical frame; correcting misperceptions about exponential growth	0.29	9.65E-06	3.57E-04
0	The sick, elderly, and immuno-compromised need our help. We all have a choice: If we go out, we risk the lives of others. But by staying home we can protect those most likely to be harmed. Stay home to protect those who are vulnerable!	paper authors	Compassion and empathy	0.29	1.57E-05	5.48E-04

Table S3: Messages that were neither more nor less persuasive than the control in merged analysis.

#	Message Text	Source	Mechanism	Beta	P-value, unadjusted	P-value, Holm-adjusted
11	Every single man, woman, and child is at great risk during the coronavirus pandemic. While older people and those with compromised immune systems are at	paper authors	Self-protection	0.19	0.0039131	1.10E-01

	greatest risk, middle aged people can also get sick and die from the virus. The overloading of the healthcare system puts us all at risk. We must act now to slow the spread of this virus.					
18	Many people carry the coronavirus without displaying symptoms. This means that we should all think and act as though we MIGHT have the virus. The best thing you can do to protect yourself and your family is to take the same precautions you would take -- staying home, washing hands -- as you would if you had the virus.	paper authors	Act like you have it	0.19	0.005228 05	1.38E-01
3	It's not only the elderly who get seriously ill from coronavirus. Young, healthy people all over the country are being hospitalized and are fighting for their lives due to the coronavirus. This virus can hurt every one of us. Stay home to protect yourself!	paper authors	Emphasi s on harm	0.16	0.013440 23	3.36E-01
23	Doctors, nurses, grocery store clerks, and others are risking their lives to keep us safe. To keep them as safe as possible, we must STAY HOME! It's our way of helping them do their jobs. Together, we can save lives and beat the virus.	paper authors	Recipro city	0.13	0.048147 41	1.00E +00
16	It is critical for us all to follow the direction of our public health officials by staying inside and avoiding contact with others when we must go out. Some people have ignored these guidelines by going on spring break trips or hosting parties, with terrible consequences for those they come in contact with. Disregarding public health guidelines isn't just irresponsible. It's also selfish and wrong, because it puts others at risk of serious illness and even death.	paper authors	Moral judgeme nt of free-ride rs	0.08	0.250537 36	1.00E +00
44	You can do your part! We can help each other in these tough times, if we all do our part we can beat COVID-19. Stay home, we can protect those who are most vulnerable by staying home, don't wait until you are directly affected. Remember you can save someone's mom, grandfather, uncle. WE CAN DO IT!	mTurk worker		0.07	0.466413 33	1.00E +00

55	We must continue to social distance and stay at home as much as possible. We are in absolute danger of having our hospitals and clinics overwhelmed and becoming infection sites for loved ones who are under medical care for cancer, stroke, or accidents. Please show you care, stay home.	mTurk worker		0.05	0.642334 37	1.00E +00
34	Our elderly population has spent their lives sacrificing and caring for us. Do your part to thank them by protecting them from the spread of the coronavirus by staying home as much as you can.	mTurk worker		0.04	0.703749 59	1.00E +00
42	Do you have COVID-19? No? Are you sure? Some people show no symptoms. Are you okay with possibly being infected and going out and infecting others? The ones you love? How would you feel if your friend got sick because of you? Or your own mother? When you think about social distancing, don't just think about yourself. Think about all the important people in your life... and losing them.	mTurk worker		0.02	0.807285 5	1.00E +00
36	We've all experienced the false alarms of pandemics in the past by our questionable media. But this time the threat is real. Covid-19 is deadly and easily contracted. Anyone you interact with can be infected even if they are not showing symptoms. This means you can easily become infected and spread the disease to your loved ones. Don't play Russian roulette: The safest thing you can do for your family and loved ones is stay home and practice social distancing. The people you love are counting on you.	mTurk worker		0.02	0.806763 77	1.00E +00
39	If someone gets the virus they are sent to the hospital alone. No family to support them. No family to sit with them. No family to say good bye if they don't make it. They are ALL ALONE no matter what their age is! ALONE! Stay home and take care of your family	mTurk worker		0.01	0.886310 66	1.00E +00
40	My daughter-in-law is a nurse and she is working very long hours trying to help her	mTurk worker		-0.02	0.857892 05	1.00E +00

	patients survive after they have gotten infected from the Coronavirus. One of her patients is a grandfather who caught the virus from his granddaughter. Stay home and protect your own family by maintaining social distance from those who are at most risk for getting infected.					
47	When you stay home, you're giving other's a fighting chance. You're giving grocery store workers time to restock the shelves and protect themselves. You're giving emergency health workers a chance to focus on individual patients. You're giving your grandma a chance to avoid the virus.	mTurk worker		-0.03	0.792922 02	1.00E +00
56	Expressing rebellion by breaking quarantine, going out for non-essentials, or socializing etc..., will not only spread the virus but will also accomplish nothing except longer shutdowns and extended quarantines for all of us. The virus does not move itself, it is moved by people. IF we stop moving so does the virus and life can get back to normal sooner.	mTurk worker		-0.03	0.743577 25	1.00E +00
9	This pandemic is a critical moment in our history. We all will look back and ask ourselves what we did in this time. Public health officials tell us that the most important thing for us to do now is stay home as much as possible, and avoid close physical contact when we must go out. Please stay home to stop the spread of this deadly virus.	paper authors	Future self-cont inuity	-0.03	0.611060 31	1.00E +00
50	I am a nurse. I have seen things no one should have to endure. Please listen and protect yourself and your loved ones by staying home.	mTurk worker		-0.09	0.379715 91	1.00E +00
13	STAY HOME, even if you don’t feel sick. Coronavirus is contagious even before you have symptoms. We all need to do this, however difficult, because it is the right thing to do: it is our duty to protect our families, friends, and fellow citizens. IT’S YOUR DUTY.	Everett et al., 2020	Deontol ogical moral frame	-0.10	0.140048 93	1.00E +00

14	STAY HOME, even if you don't feel sick. Coronavirus is contagious even before you have symptoms. We all need to do this, however difficult, because these sacrifices are nothing compared to the much worse consequences for everyone if we carry on as usual. THINK OF THE CONSEQUENCES.	Everett et al., 2020	Utilitarian moral frame	-0.10	0.12834686	1.00E+00
53	This outbreak would be over a lot sooner if we just stayed inside. If people do not listen then this is just going to get worse and prolong everything. The sooner we can stop this the sooner we can return to our lives as normal	mTurk worker		-0.11	0.2666339	1.00E+00
30	We are in a once-in-a-lifetime fight against a merciless enemy - a virus that could kill millions of people. Public health officials tell us that the key to defeating this enemy is to stay inside and avoid contact with others, so that we can keep the virus isolated. If we all do our part to contain the virus, then we can win this fight together.	Dan Greene	Conflict Mindset	-0.11	0.22674815	1.00E+00
26	If we all practice social distancing together, the virus will spread less quickly, which will reduce the burdens on our health care system.	Andy Luttrell		-0.13	0.17033643	1.00E+00
35	As the CDC and WHO say, you can be asymptomatic up to 14 DAYS! PLEASE stay home to avoid passing it to others whom it may KILL! YOU can save lives simply by staying home. Your grandparents went to war to save the world, all you have to do is stay on the couch to save the world!!	mTurk worker		-0.14	0.14040168	1.00E+00
25	If we all practice social distancing together, the virus is less likely to spread to people who are most at-risk (i.e., older adults and people with suppressed immune systems).	Andy Luttrell		-0.15	0.13775288	1.00E+00
37	Our parents and grandparents were called to war to protect us, we're being asked to stay home now to protect them. Protect your loved ones, the health care workers, by staying home.	mTurk worker		-0.15	0.12602468	1.00E+00

17	The coronavirus pandemic is a great test of our toughness. To win in this time we must be firm and we must be brave. The most effective thing most of us can do right now is to STAY INSIDE and avoid contact with others when we must go out. Making these and other sacrifices is not easy. It requires courage and grit. But we do it for our friends and our neighbors. And they do the same for us.	paper authors	Appeal to masculinity; in-group loyalty	-0.16	0.01916734	4.41E-01
22	In the fight against coronavirus, every day matters. Staying at home and avoiding social contact just one day earlier can make a great difference in the spread of the virus and can save lives. Please start social distancing NOW.	paper authors	Urgency effect	-0.16	0.01821229	4.37E-01
51	Don't risk the health and well-being of your neighbors and loved ones. Unless it's of absolute necessity, stay home to prevent the spread of the coronavirus. Our grandparents risked their lives fighting over seas; you can save lives by sitting at home on your sofa.	mTurk worker		-0.17	0.07961515	1.00E+00
1	As the threat of coronavirus builds, we as a society must act, and act together. Our efforts to prevent the spread of the virus are only as strong as the weakest link. One or two among us who fail to take this threat seriously risk harming to us all. We must stay home!	paper authors	Public good, collectivism	-0.25	0.00020559	6.78E-03
52	Take responsibility for your own actions. Your actions my cause people to get sick and die.	mTurk worker		-0.27	0.00509772	1.38E-01
48	Stopping the virus in its tracks will allow scientists working on vaccines/cures time to help as they can. Taking just one person out of the equation could help hundreds or thousands down the line. Stay home for the sake of us all.	mTurk worker		-0.29	0.00359254	1.04E-01

Table S4: Messages that were less persuasive than the control message in merged analysis.

#	Message Text	Source	Mechanism	Beta	P-value, unadjusted	P-value, Holm-adjusted
20	Over 90% of Americans are significantly changing their behavior to reduce the spread of coronavirus. Two-thirds of Americans are mostly or completely isolating themselves. Join most Americans in slowing the spread of the virus: stay home!	paper authors	Principle of social proof	-0.29	1.08E-05	3.88E-04
49	The coronavirus, aka COVID-19, knows no borders and shows no mercy. The future survival of the human race depends on you doing the right thing by staying home and self-quarantining for 14 days. You can still interact with friends and loved ones on your preferred platforms and can stay in touch with them by calling, texting or videochatting. Self-quarantine does not mean no virtual contact.	mTurk worker		-0.30	0.00232823	6.98E-02
27	Practicing social distancing helps prevent you from unknowingly transmitting the virus to who may go on to transmit the virus to even more people.	Andy Luttrell		-0.31	0.00162644	5.04E-02
46	If you don't practice social distancing you may inadvertently end up killing a close friend or family member. How could you possibly live with that? Stay home.	mTurk worker		-0.32	0.00071467	2.29E-02
32	There is nothing more important than giving your time to another person. Right now, your time spent at home social distancing is a gift of time to millions and in return those millions are gifting time to you. Let's keep everyone's clocks ticking!	mTurk worker		-0.42	1.75E-05	5.95E-04
5	As Americans we all have a patriotic duty to do whatever we can to help our country. Our nation’s public health officials tell us that the best strategy now is to stay home to slow the spread of the coronavirus. Please join together with your fellow Americans. We all must do our part by staying home.	paper authors	Patriotism moral frame	-0.48	1.54E-12	6.48E-11
45	Plenty of people have already made sacrifices to make this world the way it is.	mTurk worker		-0.49	6.29E-07	2.45E-05

	This is your time to pay it forward. Think of others because others are already thinking about you.					
38	Historians are going to look at this time and study what people did to protect themselves and others. What will you tell your children you did? If it turns out that you have coronavirus and have to tell public health officials where you've been in the past two weeks, would you be proud or ashamed?	mTurk worker		-0.61	6.65E-10	2.66E-08
21	If you leave your home for non-essential activities in many parts of the US, you can be cited for a misdemeanor. Obey the law. Stay home.	paper authors	Appeal to authority; threat of punishment	-0.63	1.04E-20	4.97E-19
10	Although times are tough right now for many Americans, we must stick together and take actions that benefit the greater good. We MUST stick together regardless of our ethnic, religious, or cultural backgrounds through these challenging times.	paper authors	Collectivism	-0.64	9.26E-22	4.63E-20
41	Be a hero! Each of us can be a hero in defeating this virus...just stay home. Keep your friends, neighbors and relatives virus free...just stay home. You can be a hero. Be a hero.	mTurk worker		-0.67	1.08E-12	4.65E-11
4	During times of war, Americans sacrifice to protect one another and support our military. Now, coronavirus has invaded our nation and we must take similar steps to protect ourselves and our fellow Americans. The most patriotic action you can take is to stay home as much as possible!	paper authors	Patriotism moral frame	-0.68	5.28E-24	2.69E-22
33	Covid-19 is the challenge of our times. Our great-grandparents fought WWII, our grandparents helped save the world from communism, our parents defeated segregation, it is time for us to rise to the occasion. Stay home!	mTurk worker		-0.73	1.63E-13	7.16E-12
31	And then the whole world walked inside and shut their doors and said: We will stop it	Facebook	Collectivism/pu	-0.74	3.83E-14	1.72E-12

	all. EVERYTHING. To protect our weaker ones. Our sicker ones. Our older ones. And nothing. NOTHING in the history of humankind ever felt more like LOVE than this.		blic goods			
2	Coronavirus is a deadly sickness. Its effects are disgusting, threatening our bodies by damaging tissue and impairing our breathing. If not stopped, this sickness will corrode our communities, infect our families, and spread beyond the control of our medical facilities. Stay home to protect yourself from this vile sickness!	paper authors	Purity moral frame	-0.77	2.80E-31	1.45E-29
54	People should begin staying at home and following the recommendations of the CDC before we lose even more of the freedoms that we as Americans cherish. No one wants martial law.	mTurk worker		-0.88	3.99E-20	1.87E-18
43	Please don't kill my family - stay home and I will too so I don't kill yours.	mTurk worker		-0.92	6.71E-21	3.29E-19
19	President Trump and the Coronavirus Task Force have ordered all Americans to avoid gatherings of 10 or more people and to stay home as much as possible. They are right! We should listen to the President: stay home!	paper authors	Appeal to authority	-0.97	4.64E-46	2.51E-44
12	As people of faith, we must join together to slow the spread of the coronavirus. This pandemic is a test of our faith and our community. We must stand by our beliefs and, most importantly, do our part to protect others. By staying home and avoiding contact when we must go out, we can slow the spread of this virus.	paper authors	Religion frame	-1.04	1.55E-53	8.65E-52
29	Practicing social distancing means complying with what government officials are demanding.	Andy Luttrell	Appeal to authority	-1.24	5.33E-38	2.82E-36
28	Practicing social distancing is what everybody else is doing.	Andy Luttrell	Social Proof	-1.38	4.09E-47	2.25E-45

The tables below show the results for pretests 1 and 2 separately.

The mixed-effects model we used was the following. The coefficient of interest is b10.
Persuasiveness(ij) = b00 + b10*MessageCondition(ij) + b20*OrderCondition(ij) + b01*PolitFactor(j) + b02*Gender(j) + b03*Age(j) + b04*Race(j) + b05*Education(j) + b06*Income(j)+ u0(j) + r(ij)

Table S5: Pretest 1 - Effects of persuasive messages, compared to a control, among full sample

Message Number	Beta	Standard Error	Unadjusted p-value	Holm adjusted p-value
0	0.343	0.109	0.002	1.92E-02
1	-0.281	0.108	0.009	9.34E-02
2	-0.997	0.109	0.000	1.43E-18
3	0.155	0.108	0.154	1.00E+00
4	-0.883	0.108	0.000	7.11E-15
5	-0.629	0.108	0.000	1.20E-07
6	0.601	0.109	0.000	5.78E-07
7	0.348	0.108	0.001	1.73E-02
8	0.483	0.109	0.000	1.33E-04
9	-0.032	0.108	0.769	1.00E+00
10	-0.885	0.108	0.000	6.66E-15
11	0.307	0.108	0.005	4.99E-02
12	-1.317	0.108	0.000	2.98E-32
13	-0.084	0.109	0.437	1.00E+00
14	-0.074	0.108	0.496	1.00E+00
15	0.594	0.108	0.000	5.86E-07
16	0.084	0.108	0.438	1.00E+00
17	-0.228	0.108	0.035	2.79E-01
18	0.261	0.108	0.016	1.42E-01
19	-1.335	0.108	0.000	4.70E-33
20	-0.387	0.108	0.000	4.86E-03
21	-0.790	0.109	0.000	7.70E-12

22	-0.136	0.109	0.211	1.00E+00
23	0.146	0.108	0.178	1.00E+00

Table S6 - Pretest 2: Effects of persuasive messages, compared to a control, among full sample

Message Number	Beta	Standard Error	Unadjusted p-value	Holm adjusted p-value
0	0.168	0.095	0.076	1.000
1	-0.257	0.094	0.006	0.221
2	-0.534	0.092	0.000	0.000
3	0.151	0.093	0.104	1.000
4	-0.409	0.098	0.000	0.001
5	-0.258	0.097	0.008	0.260
6	0.387	0.094	0.000	0.002
7	0.188	0.095	0.047	1.000
8	-0.025	0.092	0.782	1.000
9	-0.085	0.093	0.364	1.000
10	-0.328	0.095	0.001	0.022
11	-0.039	0.096	0.685	1.000
12	-0.658	0.095	0.000	0.000
13	-0.157	0.093	0.089	1.000
14	-0.213	0.099	0.031	0.924
15	0.206	0.094	0.029	0.905
16	0.023	0.096	0.813	1.000
17	-0.128	0.095	0.176	1.000
18	0.015	0.093	0.870	1.000
19	-0.418	0.097	0.000	0.001
20	-0.202	0.094	0.032	0.926
21	-0.435	0.094	0.000	0.000
22	-0.225	0.093	0.016	0.497
23	0.050	0.093	0.589	1.000

25	-0.136	0.096	0.156	1.000
26	-0.133	0.092	0.150	1.000
27	-0.303	0.097	0.002	0.066
28	-1.372	0.093	0.000	0.000
29	-1.229	0.094	0.000	0.000
30	-0.097	0.093	0.294	1.000
31	-0.731	0.096	0.000	0.000
32	-0.406	0.095	0.000	0.001
33	-0.739	0.097	0.000	0.000
34	0.037	0.092	0.684	1.000
35	-0.130	0.094	0.165	1.000
36	0.040	0.094	0.672	1.000
37	-0.129	0.095	0.174	1.000
38	-0.598	0.096	0.000	0.000
39	0.029	0.095	0.761	1.000
40	-0.014	0.095	0.879	1.000
41	-0.662	0.092	0.000	0.000
42	0.047	0.094	0.618	1.000
43	-0.905	0.096	0.000	0.000
44	0.076	0.094	0.418	1.000
45	-0.481	0.097	0.000	0.000
46	-0.316	0.093	0.001	0.028
47	-0.005	0.094	0.956	1.000
48	-0.267	0.096	0.005	0.195
49	-0.305	0.097	0.002	0.064
50	-0.063	0.096	0.510	1.000
51	-0.150	0.092	0.103	1.000
52	-0.252	0.094	0.008	0.260
53	-0.083	0.094	0.376	1.000

54	-0.888	0.094	0.000	0.000
55	0.054	0.095	0.573	1.000
56	-0.022	0.096	0.821	1.000

Compliance Filter Items, Dependent Variables, and Attention Check Questions

Pretest 1

Message ratings
How convincing do you find this reason for social distancing (staying home during the coronavirus pandemic)?
[1: Not convincing at all; 3: Not very convincing; 5: Moderately convincing; 7: Very convincing; 9: Extremely convincing]

After reading this message, how likely are you to stay home and avoid social contact for at least the next two weeks?
[1: Extremely unlikely; 3: somewhat unlikely; 5: Neither likely nor unlikely; 7: Somewhat likely; 9: Extremely likely]

Would you share this message on social media?
[1: Definitely would not share; 3: Probably would not share; 5: Maybe would share, maybe would not share; 7: Probably would share; 9: Definitely would share]

Message Creation
Please write a brief message (2-4 sentences) that you think might convince someone to follow current CDC guidelines like maintaining social distance and staying home except for essential reasons.
[Open ended]

Pretest 2

Filter question
People have many different responses to the coronavirus outbreak. Thinking about the past 24 hours, how many times did you? *{Slider scale [0 times; 24 or more times]}*
- Wash your hands
- Clean commonly used surfaces

Thinking about the past seven days, how many times did you? *{Slider scale [0 times; 15 or more times]}*
- Leave your place of residence for reasons other than food, work, medical care, and exercise

Thinking about the last time you had to leave your place of residence for any reason, how often did you observe these behaviors? *{Slider scale [0: Never; 100: Extremely often]}*

- Physically keep six feet away from others
- Wear a face mask
- Avoid touching my face

DVs

How convincing do you find this reason for social distancing (staying home during the coronavirus pandemic)?
[1: Not convincing at all; 3: Not very convincing; 5: Moderately convincing; 7: Very convincing; 9: Extremely convincing]

After reading this message, how likely are you to stay home and avoid social contact for at least the next two weeks?
[1: Extremely unlikely; 3: somewhat unlikely; 5: Neither likely nor unlikely; 7: Somewhat likely; 9: Extremely likely]

Would you share this message on social media?
[1: Definitely would not share; 3: Probably would not share; 5: Maybe would share, maybe would not share; 7: Probably would share; 9: Definitely would share]

Study 1

Filter

People have many different responses to the coronavirus outbreak. Thinking about the past 24 hours, how many times did you? *{Slider scale [0 times; 24 or more times]}*

- Wash your hands
- Clean commonly used surfaces

Thinking about the past seven days, how many times did you? *{Slider scale [0 times; 15 or more times]}*

- Leave your place of residence for reasons other than food, work, medical care, and exercise

Thinking about the last time you had to leave your place of residence for any reason, how often did you observe these behaviors? *{Slider scale [0: Never; 100: Extremely often]}*

- Physically keep six feet away from others
- Wear a face mask
- Avoid touching my face

DVs – Difference score

The coronavirus situation is changing rapidly. What you intend to do in the following days may or may not be different from what you've done in the past.

In the following questions, we'd like to learn more about **your plans for the next few days and weeks**.

In the **next 24 hours**, how many times do you **intend** to you? *{Slider scale [0 times; 24 or more times]}*

- Wash your hands
- Clean commonly used surfaces

In the **next seven days**, how many times do you **intend** to? *{Slider scale [0 times; 15 or more times]}*

- Leave your place of residence for reasons other than food, work, medical care, and exercise

The **next time you leave your place of residence**, how likely are you to: *{Slider scale [0: Extremely unlikely; 100: Extremely likely]}*

- Physically keep six feet away from others
- Wear a face mask
- Avoid touching your face

Study 2

<u>Filter</u>
Thinking about the past seven days, how many times did you? *{Slider scale [0 times; 15 or more times]}*

- Leave your place of residence for reasons other than food, work, medical care, and exercise

Thinking about the last several times you left your place of residence for any reason, how often did you do each of the following actions? *{Slider scale [0: Extremely unlikely; 100: Extremely likely]}*

- Physically keep six feet away from others
- Wear a face mask
- Wash hands or use hand sanitizer as soon as you got home

<u>DVs - Difference score</u>
The coronavirus situation is changing rapidly. What you intend to do in the following days may or may not be different from what you've done in the past.

In the following questions, we'd like to learn more about **your plans for the next few days and weeks**.

In the **next 7 days**, how many times do you **intend** to: *{Slider scale [0 times; 15 or more times]}*

- Leave your place of residence for reasons other than food, work, medical care, and exercise

The **next time you leave your place of residence**, how likely are you to: *{Slider scale [0: Never; 100: Extremely often]}*

- Physically keep six feet away from others

- Wear a face mask
- Wash hands or use hand sanitizer as soon as you got home

Study 3

Same as study 2

Attention checks

Attention check 1: used in Pretest 1 and Studies 1-3
Help us keep track of who is paying attention. Please select "Somewhat disagree" in the options below.
- Strongly agree
- Agree
- Somewhat agree
- Neither agree nor disagree
- Somewhat disagree
- Disagree
- Strongly disagree

Attention check 2: used in Studies 1-3
To ensure you took the survey seriously, please indicate one of the topics on which the survey focused.
- Voting habits
- Gender roles
- Coronavirus
- Horse racing
- Healthy diets

Message Text for Studies 2 and 3

Table S7: New message phrasing for Studies 2 and 3

Message number	Message
6	Doctors, nurses, and other health care workers are working around the clock, often risking their lives to care for patients with the coronavirus. Working long hours in highly infectious environments, many of them are falling ill.<br><br>As our health care workers put their lives on the line, we can do our part:<br>● Wear a mask when you go out.<br>● Stay at least six feet away from people outside your household.<br>● Wash your hands as soon as you get back to your home.<br><br>Our brave healthcare workers have sacrificed to help others. We should take action too.

7	A few weeks ago, Fiona was a healthy 26-year-old with no medical complications. Then she suddenly came down with a bad cough and a feeling like she could not breathe. She tested positive for COVID-19, and is now hospitalized, receiving oxygen from a ventilator, and fighting for her life.<br><br>This could be any of us. Reduce the risk to yourself and others:<br>● Wear a mask when you go out.<br>● Stay at least six feet away from people outside your household.<br>● Wash your hands as soon as you get back to your home.<br><br>If we all take these actions, we can prevent more people from suffering the way Fiona has.
15	Public health officials tell us that we must slow the spread of the coronavirus so large numbers of sick people don't overwhelm our doctors, nurses, and hospitals. If we don't slow the spread, cases will increase rapidly, suddenly spiking beyond what the health care system can handle.<br><br>We all can do our part to slow the spread:<br>● Wear a mask when you go out.<br>● Stay at least six feet away from people outside your household.<br>● Wash your hands as soon as you get back to your home.<br><br>If we take action to slow the spread now, we will save lives.
0	The sick, elderly, and immunocompromised need our help. We all have a choice: If we don't take the right actions, we risk the lives of others. But we can protect those most likely to be harmed.<br><br>We can protect those who are vulnerable by taking simple steps:<br>● Wear a mask when you go out.<br>● Stay at least six feet away from people outside your household.<br>● Wash your hands as soon as you get back to your home.<br><br>Take action to protect those who are vulnerable!
Control	Coronavirus is a respiratory illness that can spread from person to person. The virus is thought to spread mainly between people who are in close contact with one another. You can help prevent the spread of COVID-19.<br><br>We can all do our part:<br>● Wear a mask when you go out.<br>● Stay at least six feet away from people outside your household.<br>● Wash your hands as soon as you get back to your home.<br><br>These actions prevent the spread of COVID-19.

Merged Analysis of Studies 2 and 3

Groups most affected by seeing any message

In a merged analysis of studies 4 and 5, we looked at what types of people had the highest difference score after seeing a treatment message, as opposed to a control. That is, after seeing any message, they indicate that they intend to take more precautions in the upcoming days than they did in the past few days.

Gender: Women across both studies tended to have higher difference-scores than men. (M_women = 7.65, M_men = 6.08, $p < .01$ in two-sided t-test). This suggests that after seeing any message outlining key behaviors, women tend to increase their intentions to comply more than men.

Ideology: Liberals have higher difference scores than moderates, who have higher difference scores than conservatives (M_liberal = 8.33, M_moderate = 7.71, M_conservative = 5.10). All messages may be more convincing to individuals who are more liberal.

Skepticism: Part of the effects above are explained by skepticism. We divided the population into those who were highly skeptical about COVID-19 (over 50 on our scale), and those who were low in skepticism (less than or equal to 50). People low in skepticism have significantly greater difference scores than those who are highly skeptical. (M_low = 9.24, M_high = 4.45, $p < .000001$). See Table S8.

These results suggest that people who believe COVID-19 is a serious threat are most likely to be affected by reading any short message. Convincing people who are highly skeptical about the threat itself may require a more intensive intervention than reading a short message.

Table S8 - Studies 2 and 3, Compliance by Gender, Ideology, and Skepticism

**Variable**	**Category**	**Mean initial compliance**	**Mean difference score**	**Mean final compliance**	**Count**
Gender	Female	73.8	7.65	81.5	932
	Male	72.3	6.08	78.5	1196
Ideology	Conservative	72.1	5.10	77.2	953
	Moderate	70.8	7.71	83.5	400
	Liberal	75.2	8.33	83.5	775
Skepticism	Low	75.9	9.24	85.1	1028

	High	70.2	4.46	74.7	1100
